# Coordinating children’s palliative care in municipalities: a qualitative study

**DOI:** 10.1186/s12904-025-01953-6

**Published:** 2025-11-27

**Authors:** Gro Trae, Anette Winger, Marianne Nordstrøm

**Affiliations:** 1 Leve NÅ - Paediatric Palliative Care Unit, Frambu Foundation, Sandbakkveien 18, Siggerud, 1404 Norway; 2 Faculty of Health Sciences, Department of Nursing and Health Promotion, Oslo Metropolitan University, Pilestredet 32, Oslo, 0166 Norway; 3 Frambu Resource Centre for Rare Disorders, Sandbakkveien 18, Siggerud, 1404 Norway; 4 Unit for Inborn and Hereditary Neuromuscular Disorders, Department of Neurology, Oslo University Hospital, Sognsvannveien 20, Oslo, 0372 Norway

**Keywords:** Paediatric palliative care, Home-based services, Life-limiting, Life-threathening, Care coordination, Coordinator, Municipality, Qualitative

## Abstract

**Background:**

Children with palliative care conditions and their families have complex care needs. In Norwegian municipalities, designated coordinators facilitate cooperation between health and social care services to ensure a holistic approach to meeting these needs. However, information is limited concerning how coordinators perform their duties and the factors influencing their work performance.

**Aim:**

To explore coordinators' experiences and perceptions of factors influencing their work performance in relation to children's palliative care (CPC) in municipalities.

**Methods:**

Semi-structured interviews were conducted with 11 coordinators for children in palliative care and analysed using a reflexive thematic analysis approach.

**Results:**

Both internal and external factors influenced the coordinator's work performance, and they experienced a range of barriers in their efforts to achieve holistic care. Four themes were generated: ‘random knowledge on children’s palliative care’, ‘the abstract concept of coordination’, ‘striving to unite the fragmented whole’ and ‘aiming for tailored coordination’. A lack of training and experience in CPC is widespread among the coordinators. Additionally, the municipal systems seem inadequately developed to address the needs of children in palliative care and their families.

**Conclusion:**

Strengthening coordination in municipalities for children in palliative care and their families requires that coordinators receive systematic training in CPC and further development of their support systems.

**Supplementary Information:**

The online version contains supplementary material available at 10.1186/s12904-025-01953-6.

## Background

More than 21 million children on the global scale may benefit from palliative care [1], and this number increases in line with improved medical care and, consequently, prolonged survival [2]. The aim of children’s palliative care (CPC) is to improve the quality of life for children with life-limiting conditions and for their families by addressing their physical, psychological, social and existential needs [3, 4]. This holistic approach begins at the moment one becomes aware of one’s life-limiting condition and extends throughout the entire lifespan, to include bereavement support for affected family members [4, 5].

Children in need of palliative care present a wide range of conditions. While possibly curable conditions, such as cancer, constitute approximately 20% of the cases [2, 3], the majority of children eligible for palliative care have conditions that are inevitably life-limiting, with a disease trajectory that can vary significantly from days or months up to several years or that can last into adulthood [3]. The latter conditions are often rare genetic conditions, such as inborn errors of metabolism or childhood-onset neuromuscular disorders [2, 3]. Others, such as severe cerebral palsy, are imminently life-threatening due to medical complexity. Rare genetic and neurological conditions with a life-threatening or progressive disease trajectory entail comprehensive medical and social care needs, and a range of health and social care services are needed to meet these complex needs [6].

Despite the complexity, most parents of children in palliative care value staying at home with their children as much as possible [7] and they have an extensive parenting role trying to juggle caregiving tasks [8]. This demanding home care situation burdens and distresses the parents [9], and the required coordination of care to meet their child’s comprehensive care needs becomes a significant contributor to their distress [9].

In the 357 municipalities in Norway, designated coordinators are available for people in need of long-term coordinated care. These coordinators are obliged to ensure progress towards the agreed goals of care [10]. In 2022, a new statutory right to a child coordinator was enacted for children with severe health conditions who need long-term health, social and welfare services. In contrast to the traditional coordinator, the new child coordinator is anticipated to address the whole family’s needs, thereby aligning nicely with the principles of palliative care [10]. However, this new entitlement has not yet been implemented in all Norwegian municipalities. In addition, while some traditional coordinators work solely as coordinators, others have positions that combine their roles as health or social care professionals with the coordinator roles. This implies that various types of coordinators for children in palliative care currently exist within different municipalities, leading to diverse approaches in striving for successful palliative care.

Successful palliative care is characterised by trusted cooperation between families and professionals, as this enables parents to be more than just caregivers for their sick children [11]. For children needing palliative care, the municipal coordinator should ensure that the children and their families receive active and comprehensive care tailored to their unique needs [12]. To achieve such holistic care, the coordinator has a duty to interact with and catalyse cooperation among the involved health and social care workers, educational staff and other service providers involved in supporting the family. This means that the coordinator is responsible for orchestrating a large and diverse group of professionals representing different sectors and levels of care.

The role of the coordinator and care coordination are among the top identified research priorities in CPC [13, 14], and coordination of care is highly ranked as an unmet need in CPC by both parents and professionals [15, 16]. Recent studies have investigated the parents’ experiences with palliative care for children in municipalities [17]. Still, to our knowledge, no studies have explored the experiences of municipal coordinators in terms of how they perform their duties in the context of CPC. Therefore, the aim of this study is to explore the municipal coordinators’ work performance in coordinating health and social care services for children in need of palliative care. More specifically, we endeavour to answer the following research question: *What are the experiences of municipal coordinators*,* and how do they perceive factors influencing their performance in supporting children in children’s palliative care (CPC)*.

## Methodology

### Study design

The study was interpretive and explorative, using a qualitative approach and individual semi-structured interviews. We developed an interview guide for this study (see Supplementary file 1) inspired by a theoretical model of motivation and performance [18]. This model delineates how internal factors (needs, thought processes, ability and knowledge) and external factors (social facilitation, task and social factors and external dependencies) influence an employee’s motivation and work performance. The study was reported using the Reflexive Thematic Analysis Reporting Guidelines (RTARG) [19].

### Recruitment and participants

Participants were recruited through parents who had children 0–18 years of age with severe or progressive diseases, incurable genetic conditions or metabolic or neurological disorders who were receiving services from a non-hospital-based CPC unit in Norway. The parents consented to having the researchers invite their child’s coordinator in the municipality to participate in the study. Possible participants received information about the study by email or telephone and gave electronic, written consent to participate.

Participants were recruited regardless of experience, professional background and organisational setting. The only criterion for participation was that they functioned as municipal coordinators for at least one child with a life-limiting condition.

The final group consisted of 11 participants from 13 who had been requested. One rejected participation due to workload, and one did not answer the inquiry about participation. After conducting the 11 interviews, we considered the material to have satisfactory richness and diversity as this was a relatively homogenous group of participants discussing a narrow topic [20]. The geographical distribution encompasses eight out of 15 Norwegian counties allocated across all four health regions of Norway. Further demographic information on participants is listed in Table 1. The participants did not receive any reward for their participation.

**Table 1:** Demographic characteristics of participants

Characteristics	Category	*n* (%)
Gender	Women	11 (100)
Age (years)	< 40	5 (45)
	40–50	4 (36)
	> 50	2 (18)
Job title	Child coordinator	2 (18)
	Coordinator	3 (27)
	Coordinator and physiotherapist combined	5 (45)
	Coordinator and occupational therapist combined	
Education level	Higher education (> 4 years)	11 (100)
Work experience in CPC (years)	0–2	4 (36)
	3–5	2 (18)
	6–10	2 (18)
	11–15	3 (27)
Geographical coverage in Norway	Northern Regional Health Authority	1 (9)
	Central Regional Health Authority	2 (18)
	Western Regional Health Authority	1 (9)
	South-East Regional Health Authority	7 (64)

### Dataset generation

The interviews were conducted between April and June 2023 via video meetings or in a meeting room at the participant’s workplace by her choice. The interviews were audio-recorded and lasted from 45 to 65 min. Before the interview, the participants were given a summary of the definitions and goals of CPC and the study’s aim. Each interview recording was directly uploaded to a secure database at the interview’s end. The interviewer (GT) is a healthcare professional with experience from multidisciplinary work with families living with rare disorders and their health and social care providers and had no previous relationship with any of the participants.

### Data analysis

The interviews were transcribed verbatim. To analyse the interviews, we used Braun and Clarke’s reflexive thematic analysis approach [21]. In reflexive thematic analysis, interpreters actively uses their backgrounds and pre-assumptions to gain a new understanding of the research topic. The first author (GT) and last author (MN) read all interviews repeatedly to familiarise themselves with the material. Reflections and initial thoughts of themes were noted alongside familiarisation, which was helpful for interpretation and reflexivity in the analysis process. Next, GT performed inductive coding of the datasets. Both semantic and latent codes were developed from the data extracts. Initial themes were constructed by manually clustering codes together across the whole dataset. After looking through the entire material again, the themes were reviewed and refined. All authors discussed and refined the themes and the analysis report together.

### Ethical considerations

Sikt, the Norwegian Agency for Shared Services in Education and Research, has confirmed that the study complies with the Personal Data Act (Reference number 203705). Since the study did not involve collecting health-related information, approval was not required from the regional committee for research ethics. The interviewer encouraged participants to discuss their experiences in general terms and to avoid discussing the specific families through which they had been recruited. For coordinators who only followed one or a few children in palliative care, this could pose a challenge, and the interviewer was particularly mindful of this during the interviews. After each interview, participants were asked to share their feelings about answering the questions. Transcripts were de-identified and shared only with the co-authors to protect participants’ privacy. The identification key was accessible only to the first author.

## Results

Both internal and external factors influenced the coordinators’ work performance, and they experienced multiple barriers in their efforts to coordinate care as intended. These barriers left them with a sense of sole responsibility in a system unprepared for meeting children with complex care needs and their families. The results are presented below as four themes: (1) random knowledge of children’s palliative care; (2) the abstract concept of coordination; (3) striving to unite the fragmented whole; and (4) aiming for tailored coordination. For an overview of the thematic structure, see Table 2.

**Table 2:** Presentation of themes and sub-themes, descriptions of the themes and examples of quotes

**Theme**	Sub-themes		Description
Random knowledge on children’s palliative care	*Facing the unfamiliar* *Underdeveloped support systems*		The coordinators start their coordinator positions without initial training and knowledge of CPC. They also lack systematic guidance and support from their superiors.
The abstract concept of coordination			The coordinators struggle to delineate the core of coordination tasks and what their positions entail.
Striving to unite the fragmented whole	*The unprepared municipality* *Inhibiting silo working*		Many municipalities lack CPC procedures, and the coordinators uncover deficiencies in service provision. Inadequate communication flow and different legislations hamper effective coordination across levels and sectors of health and social care services.
Aiming for tailored coordination	*The value of fostering positive relationships* *Torn between different interests*		The coordinators emphasise that ‘no size fits all’ in CPC and that it is essential to actively listen to the unique family’s needs. The revealed needs of the family are not always coherent with services offered in the municipality, and coordinators can experience ethical dilemmas standing between different interests.

### Theme 1: random knowledge of children’s palliative care

The coordinators reported varying levels of expertise about CPC, both among themselves and among other health and social care professionals. This inconsistent knowledge contributed to unequal service provision for the families. The sub-themes ‘facing the unfamiliar’ and ‘underdeveloped support systems’ further elucidate this issue.

#### Facing the unfamiliar

All coordinators began their roles without initial training in CPC. Some had achieved more competence over time, but who was experienced and who was novice in working with CPC was entirely random among the participants. The lack of initial training and knowledge often left many coordinators feeling personally responsible for acquiring expertise in CPC.

I need to find things out on my own. Google is nice [laughs] But really, I need to find things out on my own. And I think that is a huge responsibility when it comes to children’s palliative care. I really do.

(Coordinator)

In many municipalities, the coordinators rarely encountered children with life-limiting conditions, which led to a lack of experience with such work tasks and sometimes a feeling of being overwhelmed. The coordinators described how they could experience a shock upon realising the severe prognosis of a child who seemed well before the onset of symptoms and that this was new to them and the municipality’s support system. They feared that their lack of expertise would lead to making mistakes or overlooking important matters.

Some coordinators had more training in CPC, arising from an earlier need for building competence, and this training improved their confidence and provided a new understanding of how to ideally perform their duties when working with the children and their families.

I have carried out a formal CPC education, which helped a lot. I did it because I was the coordinator for a child with a severe illness, and I was like, ‘What should I do?’, really, when the child was in a terminal phase. And I understood when taking this education that this is so much bigger, it is incredibly much. So, I feel that this education has contributed to knowledge about how it should be, and then you also see everything that is lacking.

(Child coordinator)

#### Underdeveloped support systems

With varied experience in CPC, the coordinators depended on the surrounding system for support and guidance. Many coordinators experienced support and an open-door policy from their leaders. Still, they missed holistic apprehension and systematic support from their superiors when providing services to the children and their families.

When we lost a child two years ago, it was like, ‘Call me if you need anything, if you want to talk’. There is no outreach offer.

(Coordinator and physiotherapist combined)

They described various understandings of how it is to work in the field of CPC among their leaders and colleagues and they wondered how other coordinators were trained and solved their work tasks. They described feeling alone and advocated more networking opportunities to share experiences with other coordinators working with CPC.

We work with the same stuff; we can lean on each other. But there is somewhat of an expectation that you need to manage this, right? We rarely talk about how tough it is for us. There is too little talk about that.

(Coordinator and physiotherapist combined)

### Theme 2: the abstract concept of coordination

In addition to the random knowledge of CPC, the coordinators also missed a clear job description and struggled to crystallise their roles as coordinators. They considered themselves to be junction points for many services and felt a massive responsibility for holding the threads. Yet, they experienced having a broad role with unclear expectations and struggled to explain their work tasks clearly. Their internal motivations for the role of coordinator would also vary, as some had actively applied for the position of coordinator, whereas others had been assigned the role as part of their profession in the municipality without the possibility of influencing the decision. Some strived to understand the concept of coordination and tried to differentiate tasks that were not considered purely coordination.

I think – I understand the need; it is chaotic for me as a child coordinator and very chaotic for the parents to keep track of everything and at the same time stand in the middle of it all. But then, what is it really to coordinate? Because it can easily tip over to thinking that you should do something, kind of be present, or do more practical tasks. But that’s not really – a child coordinator should not do that. It is the coordination that is in focus.

(Child coordinator)

Others saw their roles as being both coordinators and organisers and were desirous of doing the little extras for the families, with an inherent understanding of the parents’ demanding caregiver situations.

Maybe sometimes, it is the small things that make the big difference. They can be things I consider easy, like making an extra phone call, asking someone for something or taking part in a visit to the kindergarten […]. I consider these things as my job, but I make them content by doing it. It’s like being an extra bit on the offering side.

(Child coordinator)

The coordinators wondered if the expectations from the family and what was achievable in the support system were aligned. As they tried to figure out their roles, they found that families often had different expectations, which they thought were sometimes unrealistic. They thought such unachievable anticipations were a source of frustration and disappointment from the family’s point of view.

### Theme 3: striving to unite the fragmented whole

The coordinators had different premises for their work depending on their position, municipal structure and how developed the systems within the municipality were in terms of offering CPC services. Their mandates and experience could also influence the power balance when coordinating the team around the child and family, and they shared challenges in making services run smoothly for the best outcome for the family. This theme is described more closely in two subthemes: ‘the unprepared municipality’ and ‘inhibiting silo working’.

#### The unprepared municipality

Most coordinators experienced a lack of procedures for working with children in palliative care, and they wished that the systems were more established to take care of these families.

Our municipality has no routine or structure regarding children’s palliative care. So, the answer I get is that it has been solved from case to case without any expectations, clarifications, or defined lines on literally anything.

(Coordinator)

In some municipalities, procedures and routines for welcoming children and families were being implemented, with the coordinator actively strengthening the support system for the families. These coordinators often worked in larger municipalities with a greater number of inhabitants. Yet, accessing all the services available in the municipality could be challenging, and frequent reorganisations and staff turnover muddled this even more.

In both small and larger municipalities, coordinators described gaps between families’ needs and the services available for children and families, including even obligated services such as support for siblings or bereavement support. The coordinators found it frustrating that they couldn’t offer services that would benefit family members.

Sometimes, it’s a bit frustrating because you see their needs and want to help the parents, but then … it’s you who unfolds the needs, and then you will try to get some help, but there isn’t any; there is no help to get because no one takes on that responsibility.

(Coordinator and physiotherapist combined)

#### Inhibiting silo working

Coordinators described organisational obstacles to achieving holistic and seamless care for the families. These obstacles could be at an overarching level within the municipality, and the coordinator would question the effectiveness of the current organisation.

It’s not that the professionals don’t want to do a good job; we need to go to the level above, to the municipality and the joint responsibility of offering services of good quality, regardless of the legal circumstances. From thinking in silos to thinking holistically.

(Coordinator)

Also, between the municipality and specialist health care, the coordinators expressed the presence of some barriers, including the challenges of having a natural point of contact with specialist health care services, and they called for better care coordination within the hospitals.

I know they are supposed to have a coordinator in the specialist health care, but that’s not being so. Sometimes, I can get a contact person’s name, but they don’t function as a coordinator like … well, I know it can’t be at the same level as in the municipality, but no one has the total hold of everything in the hospital either. So, I believe it would have been easier if that coordinator function had worked better.

(Child coordinator)

Being responsible for multidisciplinary teams around the family, the coordinators described challenges in adequate communication between the municipality and specialist health care services. This was significantly linked to working digitally with individual plans for the child.

You know, continually nagging others to review, edit, update and read a document is not sustainable. While a few professionals may actively engage with it, most may not open it. As a result, it might appear well-presented, but it doesn’t truly serve its intended purpose.

(Coordinator and physiotherapist combined)

Sometimes, professionals from specialist health care services and other governmental arenas were opposed to registering on the platforms available for digital collaboration due to workplace privacy regulations. This was frustrating for the coordinators and made their jobs more cumbersome, as for example, they had to send meeting reports by regular mail. They said cooperation would have run more smoothly had everyone joined the same digital platform.

### Theme 4: aiming for tailored coordination

Regardless of their backgrounds, knowledge of CPC and ways to conform to the families, the coordinators communicated clearly that no size fits all in the context of CPC. They acknowledged that all families are unique; therefore coordinators need to be attentive, receptive, and flexible in their approaches. This was both important and challenging for the coordinators. This theme is further elaborated in two sub-themes: ‘the importance of fostering positive relationships’ and ‘torn between different interests’.

#### The importance of fostering positive relationships

The coordinators emphasised the significance of seeing the unique needs of the different families. They described the families as various, with different wishes and needs. Getting to know each family from the start was crucial, as it created a trusting relationship and tuned their work into the family’s situation.

You need to get to know them first and listen to their needs because it’s so unlike what they need. You see, I am the coordinator for 30 kids, and I don’t do it in the same way for any of them.

(Coordinator)

As part of active listening to the family’s needs, the parent’s desire for involvement could vary, thereby affecting the pace of the coordinator’s work. Some coordinators described that dealing with parents who engaged in all decisions concerning their child could delay progression in work, but they respected the parents’ wishes. At times, some coordinators would notice that the parents opted for a bit of distance during calmer periods, aiming to maintain normalcy. In such cases, the coordinators remained in the background, confident that the parents would reach out if needed.

Independent of what type of coordinator they were, the coordinators expressed an urge to do their best for the families and said they gained trust by showing action and fighting for their rights. Some described themselves as combatants for the family and were grateful for being able to take some of the burden off the families. Several valued being close to the families and having specific tasks in the family’s home, which allowed them to perceive the needs and situation of the family continuously. However, depending on their roles, the coordinators saw this as a double-edged sword, being either close to the families, (e.g. as executive physiotherapists) with less time to do coordinating work or being more distant with more time to do coordinating work.

I think a key to doing a good job is not only having these meetings when we come together to discuss the situation and ask a lot of questions, but also grasping their actual situation and their real challenges right now.

(Child coordinator)

#### Torn between different interests

While striving to offer the best support to families and deliver tailored coordination, coordinators could sometimes feel torn between meeting families’ needs and navigating municipal constraints. At times, this juggling act left them feeling inadequate for not meeting every family need, while also facing the unspoken pressure to uphold loyalty to colleagues and meet employer expectations.

I feel that the mix of roles, being part of a system that is not functioning optimally, sometimes crashes with the child’s and family’s needs. What you would like to recommend and make happen is something you know won’t … You promise gold and green forests, saying, “We need to achieve this and that,” knowing that it will not necessarily be the result.

(Coordinator and physiotherapist combined)

The coordinators expressed that their job in CPC is challenging and not for everyone. They often wanted to do more to improve each family’s situation, but struggled with limited time resources. They considered the coordination task to be something that could easily be a full-time job, at least when coordinating care for several children, and they thought it could be sufficient only to concentrate on coordination tasks. They expressed that more time for coordination tasks could help them work more proactively.

For it becomes a challenge, I sense, to measure up in all areas, to have the capacity for what is required in many of those coordination cases, to feel that one should have been more proactive and a bit more ahead of things, that it might have been a better experience for everyone if there had been the capacity to think a bit further on, so that it doesn’t turn into that hasty and more like a firefighting manoeuvre, which can occur when there is a rapid change at least.

(Coordinator and physiotherapist combined)

Along with limited time resources, the organisation of their positions and the number of children they were responsible for significantly impacted the coordinators’ ability to perform coordination tasks. Newly employed child coordinators also expressed concerns about their future resource availability.

These positions, or these resources, were created to provide high-quality services and holistic care to the families needing it the most. But that’s not possible if you have too many cases. You know, these cases … there is no exchange unless someone dies. And if a new child needs palliative care next week, you can’t put them in line waiting for another child to die before they get a coordinator.

(Child coordinator)

## Discussion

The current study provides novel insights into coordinators’ experiences and perceptions of factors influencing their performance in coordinating CPC within Norwegian municipalities. Analysis of 11 interviews resulted in the development of four themes: (1) random knowledge of children’s palliative care; (2) the abstract concept of coordination; (3) striving to unite the fragmented whole; and (4) aiming for tailored coordination. To our knowledge, this is the first study to explore municipal coordinators’ experiences when coordinating services to children in palliative care and their familes.

Due to its complexity, palliative care for children is a challenging work area for coordinators. A main finding of this study is that coordinators are often tasked with palliative care coordination without any initial training. Coordinators who later obtained a CPC education expressed that it was eye-opening to learn what the holistic approach to palliative care for children entails. Coordinators without training in CPC may gain a fragmented and incomplete understanding of CPC, resulting in inconsistent and uneven support provision to families, regardless of each family’s needs. This is consistent with concerns voiced by parents in other studies, in which they described a lack of coordination, flexibility and expertise in regional CPC services [16, 17].

In palliative care for children, three recommended levels of knowledge are required at the different levels of the support system [5]. The first level covers the basic principles of palliative care that should be used for all children, no matter where they are being cared for. The second level entails general knowledge of CPC among healthcare and social workers working in places like hospitals and general practitioners’ offices. The third level is for healthcare professionals specialising in CPC, especially those working in a dedicated CPC setting [5]. As the educational opportunities in CPC are limited in the Nordic countries, an urgent need exists for the systematic training of all coordinators working with children needing palliative care to ensure that all have at least the minimum level of knowledge as a basis for their work.

The participants’ variable positions indicated that coordinating services for children requiring palliative care within the municipality is structured in numerous ways. The subject of the rights of the child coordinator aligns with principles in palliative care for children, where the needs of the whole family’s body, mind and spirit should be addressed [5]. Providing families with a child coordinator would therefore be beneficial in reaching the goals in palliative care. However, merely acquiring a child coordinator will not be sufficient if the coordinator is not trained in CPC or delegated with the authority to make decisions. To ensure equity in service provision for families, municipalities must enhance their systems regarding their child coordinators to promote greater consistency in delivering services to children with complex palliative care needs.

In a scoping review by Boyden et al. [22], parents ranked care coordination as one of the most critical domains in home-based palliative care for children, and the need for coordinated care is well emphasised in international CPC guidelines [5]. In Norway, the guidelines state what the coordinators are responsible for, but no specific descriptions are provided regarding how they should perform their work tasks. Without such clear job descriptions, a lack of standardised approach to care coordination will likely persist for children with life-limiting conditions and their families.

Our study shows that coordinators experience their mandate as blurry, they feel insecure about their roles, and their expectations are not clarified. These findings coincide with another Norwegian study exploring the experiences of coordinators for children with disabilities; that study found that the coordinator role is emeshed with challenges that include role understanding, resource access, job scope and professional background [16]. Clarifying the work tasks of coordinators for all children with complex care coordination needs could ensure more equal delivery of services to these children, independent of the severity of the child’s condition.

Another finding in our study is the existence of discrepancies between the family’s needs, as identified by the coordinator, and the lack of municipality-provided services, such as support for siblings and bereavement support. This places the coordinators in a difficult position between family and employer when aiming to ensure holistic and family-centred care. Dealing with conflicting interests—alongside feelings of inadequacy due to insufficient knowledge, unclear expectations, and limited resources—makes the coordinators vulnerable to compassion fatigue, moral stress, and burnout [23]. Municipalities should establish sufficient and systematic support systems to ensure robust and sustainable care coordination. Regular networking with other coordinators involved in CPC would be beneficial for discussing dilemmas and sharing experiences which is also important for developing a professional environment in CPC.

Our study also reveals challenges in communication flow and information exchange between systems within the municipality and between municipality services and specialist health care. In previous research, CPC providers and parents have highlighted the necessity for enhanced planning and coordination during the transition from hospital to community services [16, 24]. The absence of such planning and collaboration across organisational boundaries may lead to fragmented care coordination and disclaimers, leaving the families in limbo when returning home and alone to sholder the responsibility for reaching out to required services.

The coordinators in our study wished the municipality was better prepared for welcoming children in palliative care and their families. Staying at home as much as possible is valued by parents [7] and important for gaining a sense of normalcy [25]. Further, home-based palliative care may improve quality of life and reduce symptom burden for the children [26]. To be adequately prepared and equipped, the specialist healthcare and the municipalities need close and continuous communication. Previous research has demonstrated that use of electronic health systems can improve communication between health care personnel and increase family empowerment in CPC [27]. Some coordinators in our study stated that electronic health systems exist for collaboration across sectors and levels in health and social care services but that these opportunities for digital collaboration are not being fully utilised. Utilising digital collaboration opportunities and establishing natural points of contact may facilitate easier and earlier sharing of information about the child’s illness trajectory between hospitals and municipalities. This, in turn, could assist coordinators in preparing for the family’s return home in advance.

Lastly, the coordinators in our study highlighted that a trusting relationship between the coordinator and the family and capturing the unique family’s needs are fundamental for constructive collaboration and support. A need to be close and connected to the family is also a finding in a scoping review on the needs of healthcare personnel providing home-based CPC [28]. To achieve this, coordinators require enough time and inner drive for their duties. As continuous monitoring of the family’s needs is recommended in CPC guidelines [5, 12], the use of systematic methods might be beneficial to aid the coordinators in mapping and addressing the needs of each family. Future research might look at interventions that coordinators can utilise for this purpose. By focusing on addressing the family’s needs to achieve the best possible quality of life for all family members, coordinators can create an environment of trust and teamwork, thereby improving care for children with life-limiting conditions and their families.

## Strengths and limitations

A strength of this study is the recruitment procedure, in which the selection was random in terms of geographical distribution, types of coordinators and coordinator experience. This gave valuable insights into how these coordinators experience their work situations. Nevertheless, these were only 11 of the many coordinators in the Norwegian municipalities, and all coordinators may not recognise the experiences of this small group. Differences might also exist between coordinators who voluntarily apply for positions as coordinator and other professionals who are assigned the role, and our study does not uncover these. Another noteworthy point is that researchers with certain preconceptions conducted this study, whereas others reseachers might have interpreted the interviews differently. Through openness and reflexivity, we aim to gain trustworthiness and credibility.

## Conclusion

In the municipalities, coordinators are motivated to provide optimal support for the child and family. However, they face challenges due to a lack of initial training and articulated work instructions, procedures and routines. As a result, the coordinators’ work is performed in various ways. Striving for the best outcome for families is challenging for coordinators working in a system with multiple limitations. A need exists to enhance both the external framework conditions and the coordinators’ inner skills, knowledge and training in CPS to facilitate improved care coordination for children in palliative care and their families. This can be accomplished by developing educational resources for coordinators and their leaders and enhancing municipal CPC services by elaborating on procedures and defining the coordinators’ work tasks.

## Supplementary Information

Supplementary Material 1.

## Data Availability

Due to privacy regulations, the datasets generated and/or analysed during the current study are not publicly available but are available from the corresponding author upon reasonable request.
