# Correction: Coordinating children’s palliative care in municipalities: a qualitative study

**DOI:** 10.1186/s12904-026-02010-6

**Published:** 2026-02-11

**Authors:** Gro Trae, Anette Winger, Marianne Nordstrøm

**Affiliations:** 1 Leve NÅ - Paediatric Palliative Care Unit, Frambu Foundation, Sandbakkveien 18, Siggerud, 1404 Norway; 2 Faculty of Health Sciences, Department of Nursing and Health Promotion, Oslo Metropolitan University, Pilestredet 32, Oslo, 0166 Norway; 3 Frambu Resource Centre for Rare Disorders, Sandbakkveien 18, Siggerud, 1404 Norway; 4 Department of Neurology, Unit for Inborn and Hereditary Neuromuscular Disorders, Oslo University Hospital, Sognsvannveien 20, Oslo, 0372 Norway

**Correction: BMC Palliat Care 25**,** 2 (2026)**

**https://doi.org/10.1186/s12904-025-01953-6**

Following publication of the original article [1], the author reported that an incorrect Table 2 has been included in the published article. The table was from their other article.

Below is the incorrect Table 2.

**Table 2:** Presentation of themes and sub-themes, descriptions of the themes and examples of quotes

Theme	Subthemes	Despription	Excerpts of quotes
Nutrition is a core component of comprehensive palliative care	Nutritional management requires a multidisciplinary approachThe goals of nutritional care warrant ethical discussionsNutritional management impacts quality of life	Dietitians regard nutritional care as a responsibility best managed through an interdisciplinary approach rather than solely by the dietitian. The joint responsibility should also address the many ethical concerns in the field, as nutritional care impacts quality of life.	‘Because it’s also essential, just like with cardiopulmonary resuscitation, to consider these decisions in advance’
Organisational placement affects the dietitian's involvement.	The dietitian’s areas of responsibility influence their capacity to work with children’s palliative careOrganisational structure either promotes or hinders multidisciplinary collaboration	The dietitians indicate that the organisational structure plays a crucial role in shaping their ability to collaborate effectively with other professionals regarding nutritional management for children in palliative care.	‘Nutrition is often a bit... well, nurses and doctors often work closely together, and because I’m not permanently employed there, […] I’m on the sidelines’
Fragile structures for nutritional care	Limited basic knowledge and access to informationDietitians as guardians of nutritional careNon-systematic collaboration with municipality-based services	Dietitians often lack reliable clinical decision support and guidelines for clinical decision-making and must ensure that nutrition is prioritised within the multidisciplinary team. They rarely collaborate with municipality-based services closer to the families.	‘And then we sort of have to knock on the door and say that this needs to be interdisciplinary, and we need to take a broader perspective on it. I can’t be the only one responsible for that part’
Close bonds between the family and the dietitian.	Adjusting to meet the family’s needsGently pushing in the recommended direction	Dietitians maintain close, continuous contact with families, adapting their approach to meet the child's evolving needs as their condition progresses.	‘Therefore, when we notice that it’s becoming difficult and there’s a risk of aspiration, we must discuss it with the parents, explain the situation, and allow them the time they need to process it’

Below is the correct Table 2.

**Table 2:** Presentation of themes and sub-themes, descriptions of the themes and examples of quotes

Theme	Sub-themes	Description	Excerpts from related quotes
Random knowledge on children’s palliative care	Facing the unfamiliarUnderdeveloped support systems	The coordinators start their coordinator positions without initial training and knowledge of CPC. They also lack systematic guidance and support from their superiors.	‘I need to find things out on my own. And I think that is a huge responsibility when it comes to children’s palliative care, I really do’.‘We rarely talk about how tough it is for us. There is too little talk about that’.
The abstract concept of coordination		The coordinators struggle to delineate the core of coordination tasks and what their positions entail.	‘But then, what is it really to coordinate? Because it can easily tip over to thinking that you should do something, kind of, be present, or do more practical tasks. But that’s not really.. a child coordinator should not do that. It is the coordination that is in focus’.
Striving to unite the fragmented whole	The unprepared municipalityInhibiting silo working	Many municipalities lack CPC procedures, and the coordinators uncover deficiencies in service provision. Inadequate communication flow and different legislations hamper effective coordination across levels and sectors of health and social care services.	‘Regarding children’s palliative care, our municipality has no routine or structure’.‘We need to go to the level above, to the municipality and the joint responsibility of offering services of good quality, regardless of the legal circumstances. From thinking in silos to thinking holistically’.
Aiming for tailored coordination	The value of fostering positive relationshipsTorn between different interests	The coordinators emphasise that ‘no size fits all’ in CPC and that it is essential to actively listen to the unique family’s needs. The revealed needs of the family are not always coherent with services offered in the municipality, and coordinators can experience ethical dilemmas standing between different interests.	‘You need to get to know them first and listen to their needs because it’s so unlike what they need’.‘I feel that the mix of roles, being part of a system that is not functioning optimally, sometimes crashes with the child's and family's needs’.

The original article has been updated.

## References

[1] Trae G, Winger A, Nordstrøm M. Coordinating children’s palliative care in municipalities: a qualitative study. BMC Palliat Care. 2026;25:2. 10.1186/s12904-025-01953-6. 10.1186/s12904-025-01953-6 PMC12763916 41310656

