# Supplementary material for: Coordinating children’s palliative care in municipalities: a qualitative study
Source: BMC Palliat Care. 2025 Nov 27;25:2. doi: 10.1186/s12904-025-01953-6 (PMC12763916; doi:10.1186/s12904-025-01953-6)
Supplement: Supplementary file 1 — Supplementary Material 1. [file 12904_2025_1953_MOESM1_ESM.docx]

**Supplementary file 1**. Interview guide

**Municipal coordinators’ work performance within children’s palliative care**

**Introduction:**

Give the presentation and short briefing about children’s palliative care (CPC) and examples of diagnoses. Explain the topics of the interview and how it will be used (thoughts about coordinating work with children in palliative care, the coordinator’s role and the performance of work tasks) and how it will be used.

**Practical information:**

The interview will last for around one hour. All information will be de-identified, and the participant will not be recognisable. The interviewer has a duty of confidentiality. Nevertheless, the participant still has the right to withdraw from the study.

| **INTERNAL FACTORS** | **Main questions** | **Promts** |
| --- | --- | --- |
| Knowledge | Can you start by telling me about your work with children with life-limiting or life-threatening conditions?  How do you experience your own knowledge about following-up on children in palliative care?  How did you acquire this knowledge?  How do you experience the CPC knowledge of other professionals who work closely with children in palliative care? |  |
| Needs | What are your thoughts on the coordination of services for children with life-limiting or life-threatening conditions?  What do you think about how the coordinating work for this group *is*?  How do you think it *should be*?  What role do you have in following-up with children in palliative care?  Do you experience ethical dilemmas in your work? What are the dilemmas? | Loyalty to family vs. loyalty to employer |
| Goals | As a coordinator, what do you think is the best way to work multi-disciplinarily with children in palliative care?  How is the coordinating work concerning CPC organised?  What routines are in place? | Talks with relatives, colleagues or team; department meetings  Personal assistants (activity and participation) or home nursing (health care) |
| **EXTERNAL FACTORS** |  |  |
| Practical arrangements | Can you tell me about your tasks related to being coordinator?  How is your position anchored in the municipality?  How do you get in touch with new families?  Does this affect your availability for the children in palliative care?  What are your experiences with digital platforms and tools for collaboration? | Sole position or combined positions  Before families return home or after some time at home |
| Social facilitation | What other professionals do you collaborate with in relation to your coordinating work?  How do you experience the follow-up from your leader regarding your work performance?  Do you collaborate with any paediatric palliative care teams?  Do you experience coordinating services for the child is being treated as a priority? |  |
| Reward | Can you tell me how your efforts to coordinate services within CPC are acknowledged?  Is your role as a coordinator in professional discussions valued?  Do the families express that they feel the job you perform is useful? |  |
| **STANDARDS/**  **GUIDELINES** |  |  |
|  | Do you follow any guidelines in your work with children in palliative care? What guidelines? |  |

**Closing:**

What was it like to answer these questions?

[Provide a short summary of the main points]. Is there anything that you would like to add?

I have now finished my questions. Is there anything more you would like to add before we end the interview?

Thank you for your participation. [Remind the participant that all information will be de-identified].
